# The Cassidinae beetles of Longnan County (Jiangxi, China): overview and community composition

**DOI:** 10.3897/BDJ.7.e39053

**Published:** 2019-10-18

**Authors:** Peng Liu, Chengqing Liao, Jiasheng Xu, Charles L. Staines, Xiaohua Dai

**Affiliations:** 1 Leafminer Group, School of Life Sciences, Gannan Normal University, Ganzhou, China Leafminer Group, School of Life Sciences, Gannan Normal University Ganzhou China; 2 Smithsonian Environmental Research Center, Edgewater, United States of America Smithsonian Environmental Research Center Edgewater United States of America; 3 National Navel-Orange Engineering Research Center, Ganzhou, China National Navel-Orange Engineering Research Center Ganzhou China

**Keywords:** Cassidinae, Hispini, Cassidini, host plant, Longnan County

## Abstract

There are few reports on the community composition and diversity pattern of the Cassidinae species of China. Compared to the neighbouring provinces of Guangdong, Fujian and Zhejiang, the Cassidinae richness in Jiangxi Province is under-reported. Longnan City, a biodiversity hotspot in Jiangxi Province, was chosen to obtain the first overview of the Cassidinae beetles. The sample coverage curves for the three sample sites reached an asymptote which indicated sampling was sufficient for data analysis. A total of eight tribes, 16 genera, 59 species and 1590 individuals of Cassidinae beetles were collected. Most belonged to the tribe Hispini (1121 individuals; 70.5%), followed by the tribe Cassidini (161 individuals; 10.13%) and the tribe Oncocephalini (159 individuals; 10.0%). The remainder (149 individuals) belonged to five tribes (Gonophorini, Basiprionotini, Callispini, Notosacanthini and Aspidimorphini). The tribes Notosacanthini, Aspidimorphini and Oncocephalini were newly recorded for Jiangxi Province. There were 14 families, 27 genera and 39 species of host plants of Cassidinae beetles in Longnan County. Cassidinae larvae mainly feed on the plant families Poaceae, Rosaceae, Lamiaceae and Rubiaceae. Most host-plant associations are new reords for the beetle species. This research, together with our planned future work in China, may help to explain the geographical distribution, diversity patterns and host plant associations of these beetles.

## Introduction

With more than 6000 species, Cassidinae s. l. is the second most diverse subfamily of Chrysomelidae (Chen et al. 1986, Flinte et al. 2009b, Liao et al. 2015, Staines 2015, Borowiec and Świętojańska 2019). The subfamily consists of the hispine beetles (Hispinae s. str.) and the tortoise beetles (Cassidinae s. str.) (Staines 2002). Cassidinae are widely distributed, but are most abundant in the tropical and subtropical regions of South America (Chaboo 2007). Cassidinae show strong adaptability in their host plants; for example, the leaf-mining Hispines feed on more than 80 families and 800 species (Liao et al. 2015). Some species of Cassidinae are important agricultural and forestry pests (Chen et al. 1986). *Dicladispa armigera* (Olivier) was a primary pest on rice, *Oryza sativa* L., in south-eastern China (Chen et al. 1986, Li 1990); *Dactylispa setifera* (Chapuis) was a major pest of corn, *Zea mays* L., in Guangxi in 1960s-1970s, as well as in the 1990s (Chen et al. 1986, Zhang and Lu 1990); *Platypria melli* Uhmann has severely damaged the leaves of Rhamnaceae fruit trees *Hovenia acerba* Lindl. and *Ziziphus jujuba* Mill. (Chen et al. 1986, Liao et al. 2014); *Cassidispa relicta* Medvedev is a severe threat to the dominant trees, *Betula platyphylla* Sukatchiev and *Ulmus pumila* L., in Inner Mongolian forests in recent years (Liao et al. 2018b). Invasive Cassidinae species such as palm-feeding *Brontispa longissima* (Gestro) and *Octodonta nipae* (Maulik) are substantial threats to economic crops and native plants (Peng et al. 2018, Zou et al. 2019).

Before the 1960s, the species of Cassidinae occurring in China were identified and reported by foreign taxonomists, including Baly J.S., Boheman C.H., Gestro R., Gressitt J.L., Hincks W.H., Kimoto S., Maulik S., Spaeth F., Uhmann E. and Weise J. (Chen et al. 1986, Sekerka et al. 2016). Since the 1960s, Chinese entomologists have reported many new species and records (Chen et al. 1986). However, only a few systematic monographs on the Chinese Cassidinae fauna at either a whole-country scale or regional scale have been published since the 1950s (Gressitt 1950, Gressitt 1952, Gressitt and Kimoto 1963, Chen et al. 1986, Kimoto and Takizawa 1997, Lee and Cheng 2007, Lee and Cheng 2010, Lee et al. 2016, Qi 2009). In 1963, there were 38 genera and 205 species in China (Gressitt 1950, Gressitt 1952, Gressitt and Kimoto 1963, Chen et al. 1986). In 1986, the numbers increased to 49 genera and 417 species (Chen et al. 1986). Currently, over 500 Cassidinae species have been reported in China (Kimoto and Takizawa 1997, Hua 2000, Borowiec and Sassi 2002, Świętojańska and Borowiec 2006, Lee and Cheng 2007, Lee and Cheng 2010, Lee et al. 2016, Lee et al. 2009, Lee and Sekerka 2018a, Lee and Sekerka 2018b, Lee 2015, Lee 2009, Lee and Staines 2010, Lee et al. 2011, Lee et al. 2012, Borowiec and Lee 2008, Borowiec and Lee 2009, Borowiec 2009, Aston 2009, Qi et al. 2008, Qi 2009, Staines 2015, Borowiec and Świętojańska 2019, Liao et al. 2018b, Świętojańska 2001). However, according to our collecting records in recent years, China should have higher Cassidinae richness than reported.

Most studies on Cassidine species occurring in China focus on morphological descriptions, with some with biological information including host plant records and genome composition (Chen et al. 1986, Lee et al. 2009, Qi 2009, Guo et al. 2017a, Guo et al. 2017b, Yang et al. 2017, Yao et al. 2017, Liao et al. 2018a, Liao et al. 2018b, Liu et al. 2018, Peng et al. 2018, Xu et al. 2018), but almost none on community composition and diversity patterns (Chen et al. 1986). Moreover, it was unfortunate for Cassidinae that urbanisation and agricultural activities increased anthropogenic disturbances, which have high negative impacts on their distribution, diversity and dynamics (Nummelin and Borowiec 1992, Ghate et al. 2003, Chaboo 2007, Sánchez-Reyes et al. 2019). Many Cassidinae species had disappeared before being documented. Some natural forests were destroyed for the establishment of economic plantations, for example, *Hevea brasiliensis* (Willd. ex A.Juss.) Müll.Arg., *Eucalyptus* spp. and *Citrus sinensis* (L.) Osbeck, which are marked threats to Cassidinae biodiversity, especially in southern Chinese provinces of Yunnan, Guangxi and Jiangxi (Dai et al., personal observation). Six tribes, 12 genera and 49 species of Cassidinae beetles have been reported in Jiangxi Province (Chen et al. 1986, Zhang et al. 1987). But, However, no particular site in Jiangxi Province has been thoroughly inventoried and no single study has looked at the diversity pattern at different taxonomic levels. Compared to the neighbouring provinces of Guangdong, Fujian and Zhejiang, the Cassidinae richness in Jiangxi Province is under-reported, especially for endemic species (Chen et al. 1986). Since 2012, our Leafminer Group at Gannan Normal University has discovered many new records of Cassidinae species and their host plants in Jiangxi and many other provinces in China (Dai et al. unpublished data). There has been no systematic analysis on the faunal composition and diversity pattern of the Cassidinae beetles in Jiangxi.

The Nanling Mountains are a critical biogeographical line between the mid-subtropical and the south-subtropical zones in China (Zeng et al. 2018). Nanling is also one of the KBAs (Key Biodiversity Areas) in China (Huang et al. 2012, Zhang et al. 2014). Located at the north slope of Nanling Mountains, Longnan County is a representative biodiversity hotspot in Jiangxi Province (Dai et al. 2014, Dai et al. 2013, Dai et al. 2019, Bai et al. 2015, Bai et al. 2016, Liu et al. 2002), with one national nature reserve, six county nature reserves, one national forest park and three provincial forest parks. This research aimed to provide a first overview and a quantitative species list of Cassidinae, estimate their community composition and understand which tribes, genera and species were the most diverse in Longnan County. Our research also could benefit from knowing the effect of human activities on forest biodiversity and providing some information for habitat management and pest control.

## Material and methods

### Collecting sites and habitats

The insects and their host plants were collected in Longnan County from 2012 to 2018. Located in the southern tip of Jiangxi Province and in the transitional area between the mid subtropical zone and the southern subtropical zone, Longnan has a subtropical monsoon climate with an annual average temperature of 18.9℃ (-6℃ - 37.4℃), an annual rainfall of 1020.8-2595.5 mm and four distinct seasons. Spring (March-May) is warm and rainy, Summer (July-August) is hot and humid, Autumn (September-November) is cool and dry, while winter (December-February) is dry and slightly cold (Dai et al. 2019, Liu et al. 2002).

**Figure 1.**
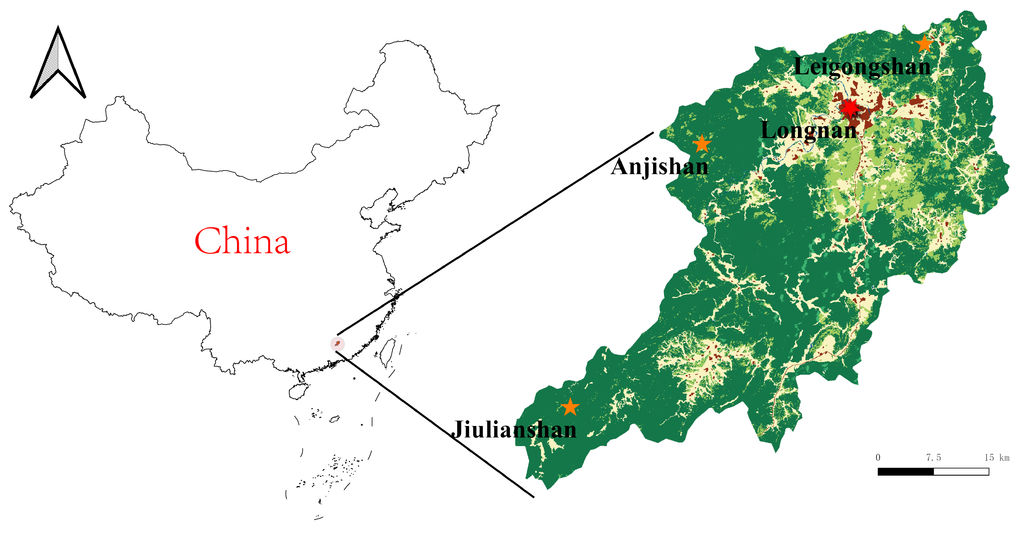
Map of Longnan County and locations of three sample sites.

Three different sites were explored (Fig. 1). These sites are exposed to different degrees of human influences, which is reflected in the quantity of resident population, road network and natural vegetation coverage in the area.

- Jiulianshan National Nature Reserve (JLS) (Fig. 2a, b): A 13,411.6 hm^2^ national nature reserve (24.58°N, 114.45°E) that is approximately 80 km from Longnan County Town. With forest coverage of 94.7%, the main vegetation type is primary subtropical evergreen broad-leaved forests, with a high diversity of leaf-mining insects (Bai et al. 2015, Bai et al. 2016, Dai et al. 2014, Dai et al. 2013, Dai et al. 2019). The human disturbance level is the lowest amongst the three sites. Collecting months and years were January (2013, 2014, 2016, 2017, 2018), February (2013), March (2013, 2014), June (2012), July (2012, 2016, 2017, 2018), August (2012, 2014), November (2014) and December (2014). Five tribes, six genera and seven species are previously reported at JLS (Liu et al. 2002, Zhang et al. 1987).
- Anjishan Provincial Forest Park (AJS) (Fig. 2c, d): A 6,093 hm^2^ provincial forest park (24.87°N, 114.61°E) that is approximately 30 km from Longnan County Town. The main vegetation types are secondary evergreen broad-leaved forests, economic bamboo forests and economic coniferous forests. The human disturbance level is intermediate. Collecting months and years were January (2018), April (2014, 2017), May (2014, 2017, 2018), June (2015), July (2014, 2015, 2016, 2017, 2018), August (2017), September (2018), October (2016) and November (2015, 2016). No Cassidinae species has been reported at AJS.
- Leigongshan Family Farm (LGS) (Fig. 2e, f): A 28-hm^2^ family farm (24.98°N, 114.88°E) that is approximately 10 km from Longnan County Town. The main vegetation types are Chinese fir forests and orange orchards. The human disturbance level is the highest. Collecting months and years were April (2016), June (2014, 2015), July (2015, 2016, 2017), August (2018) and November (2014). No Cassidinae species has been reported at LGS.

### Sampling techniques

Cassidines were located by visual inspection (generally from 08:30 h to 15:30 h) of plants by looking for adult or larval feeding damage. Adults, larvae or pupae, as well as their host plants, were manually collected and placed in plastic zip-lock bags (28 cm × 40 cm) and the collection location and date were recorded. Some Cassidinae larvae or pupae were reared to adults in the laboratory. Most adults were pinned (1095 individuals) and others were preserved in 100% ethanol at -80℃ (495 individuals). All specimens are deposited in our laboratory at Gannan Normal University (25.80°N, 114.89°E), which is approximately 130 km from Longnan County Town.

Samples of every habitat were collected along representative investigation routes at each site (10 routes for JLS, 5 for AJS and 2 for LGS), established according to the habitat preferences of Cassidinae beetles. We assumed that sufficient samples had been collected when the sample coverage curve reached an asymptote.

Adults were identified to the species/genus/family levels except two unknown species using keys ([Bibr B5302791][Bibr B5302762], Staines 2012) under a stereoscopic microscope. Photos of Cassidinae beetles were taken with Canon EOS 7D and Olympus stereomicroscope SZX16 to aid in identifcation and to document the species, as in Liao et al. (2018a), Liao et al. (2018b). Host plants were confirmed by either larval or adult feeding damage. In the laboratory, the plants and damaged leaves were individually scanned using an Epson 10000XL scanner, as in Dai et al. (2018). Plant species were identified by Prof. Renlin Liu (Gannan Normal University) and Mr. Chao Fu (Gannan Normal University).

It was not possible to identify all beetles to the species-level. These Cassidinae were identified to the genus-level and included in the data analyses.

### Data analyses

After the identification of all collected specimens, the data were input to Microsoft Excel 2016 for analysis. The land cover data of Longnan County were obtained from GlobeLand30 (http://www.globallandcover.com) for the year 2010 (Chen and Chen 2018). The map was produced with QGIS 3.8 (QGIS Development Team 2019). Sample coverage analyses were performed with iNEXT Online (https://chao.shinyapps.io/iNEXTOnline) (Hsieh et al. 2016).

All data used in our analyses were available in Suppl. materials 1, 2.

## Results

### Sample coverage

**Figure 3.**
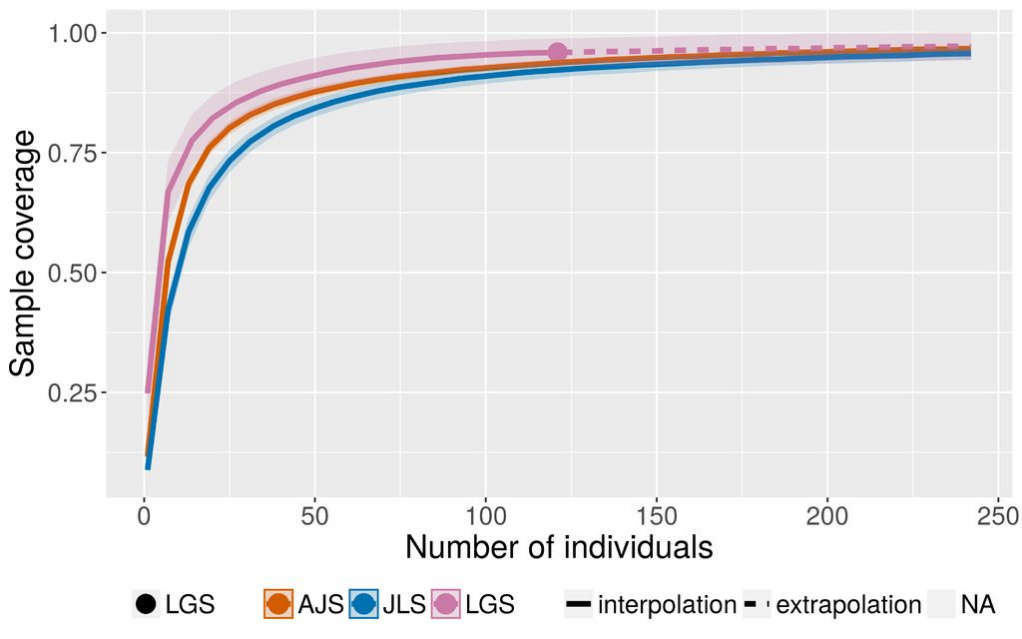
Sample coverage curves for Cassidinae collections in Longnan County.

The sample coverage curves of Cassidinae beetles at three sample sites showed an upward trend which inclined towards stability and the sample coverage of all three sites are close to one (Fig. 3). The results indicated that the sampling effort was sufficient for faunal composition analyses.

### Faunal composition of Longnan Country

All the individuals were identified to species except four individuals which were identified to genus (Suppl. material 1). A total of eight tribes (Figs 4, 5), 16 genera, 59 species and 1590 individuals of Cassidinae were collected from the three sites. Most beetles belonged to the tribe Hispini (1121 individuals; 70.5%), followed by the tribe Cassidini (161 individuals; 10.13%) and the tribe Oncocephalini (159 individuals; 10.0%). The rest (149 individuals) belonged to five tribes (Gonophorini, Basiprionotini, Callispini, Notosacanthini and Aspidimorphini). This is the first record of the tribes Notosacanthini, Aspidimorphini and Oncocephalini in Jiangxi Province.

**The Hispini**

**Figure 6.**
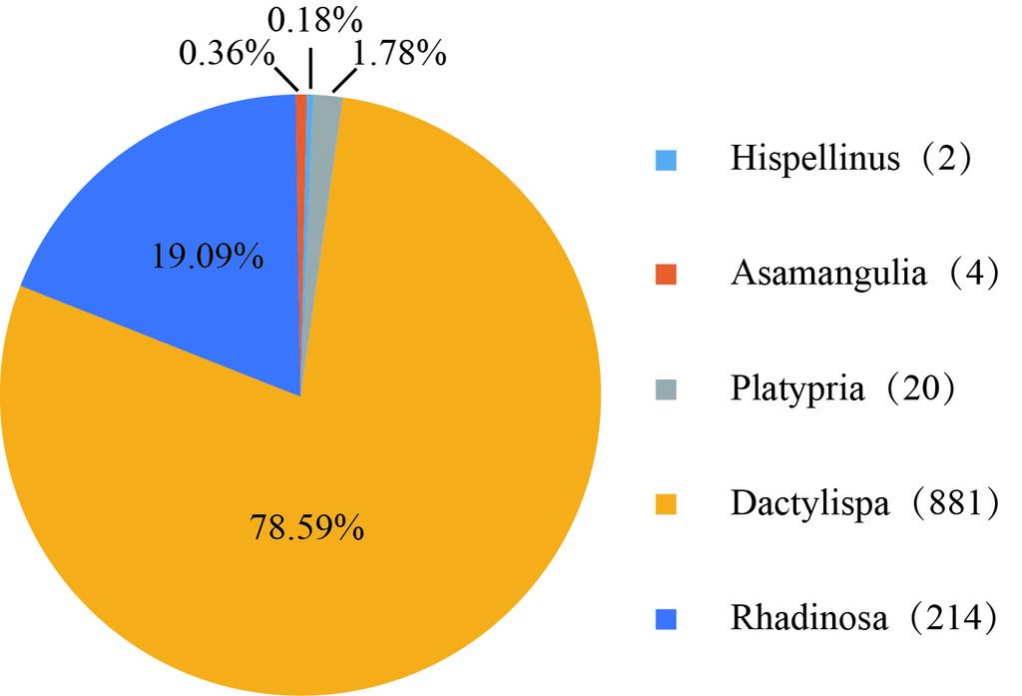
Percentage distribution of the Hispini individuals into the five genera.

Individuals belonged to five different genera: *Hispellinus*, *Asamangulia*, *Platypria*, *Dactylispa* and *Rhadinosa* (Fig. 6). Most Hispini belonged to the genus *Dactylispa* (881 individuals; 78.59%). Other genera were *Rhadinosa* (214 individuals; 19.09%), *Platypria* (20 individuals; 1.78%), *Asamangulia* (four individuals; 0.36%) and *Hispellinus* (two individuals 0.18%).

**Figure 7.**
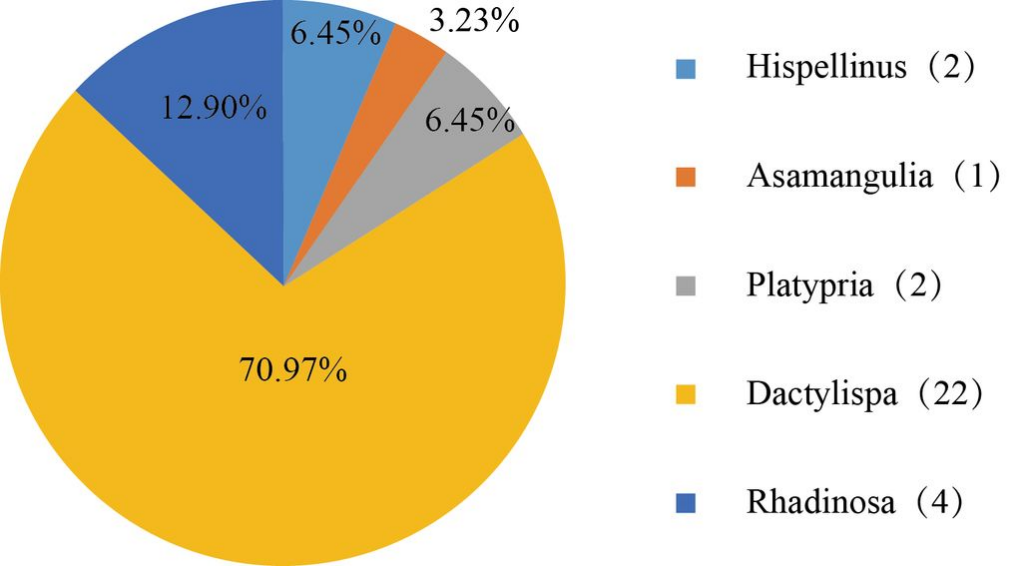
Percentage distribution of the Hispini species into the five genera.

There were 32 species of Hispini collected in Longnan County (Fig. 7), with one species of *Asamangulia*, two species of *Hispellinus* and two species of *Platypria*. The most abundant genus was *Dactylispa* (70.97%, 22 species), followed by *Rhadinosa* (12.90%, four species, including two unidentified species). At species level, *Dactylispa paucispina* Gressitt was the most common species (192 individuals).

**The Cassidini**

**Figure 8.**
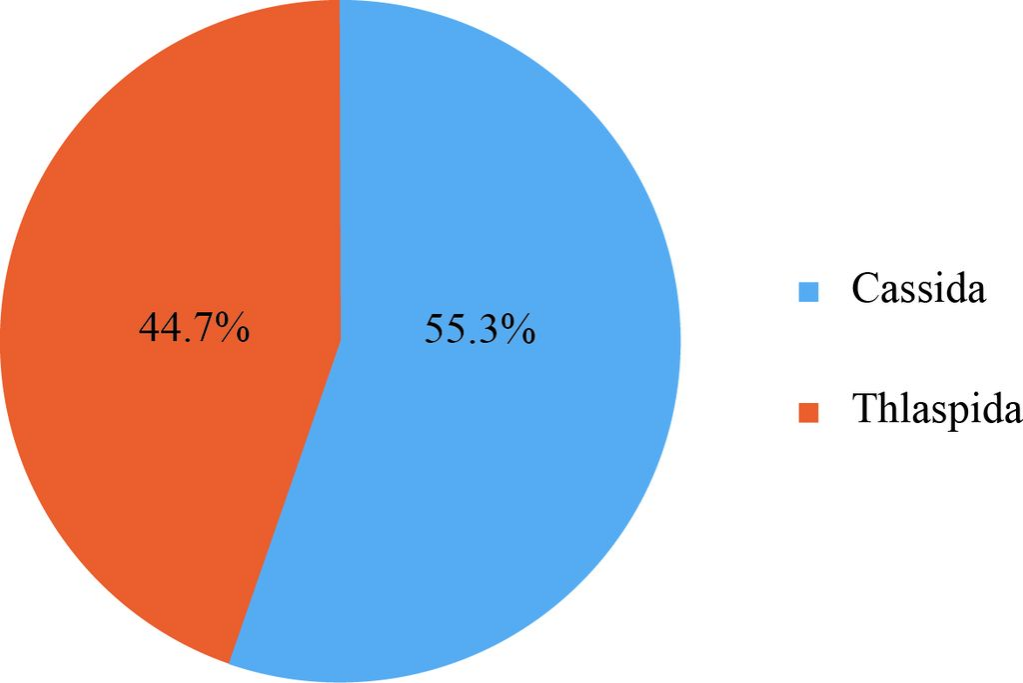
Percentage distribution of the Cassidini individuals into the two genera.

The Cassidini specimens belonged to two genera (*Cassida* and *Thlaspida*) (Fig. 8). *Cassida* had 89 individuals (55.28%), while the *Thlaspida* had 72 individuals (44.72%).

*Cassida* was the dominant genus in both species richness and individual number in the Cassidini. Cassidini had nine species, with eight species belonging to *Cassida* and one species to *Thlaspida*.

**Figure 9.**
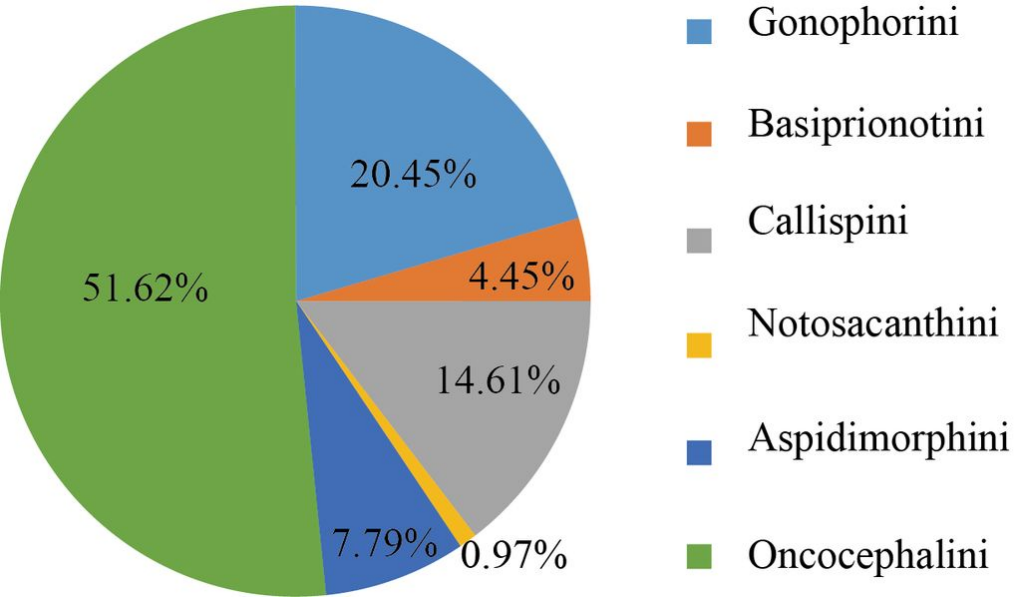
Percentage distribution of the Cassidinae individuals except for Hispini and Cassidini.

In the tribes other than Hispini and Cassidini, the Oncocephalini had the largest numbers of individuals (159 individuals) (Fig. 9), followed by Gonophorini (63 individuals). The least abundant tribe was Notosacanthini (three individuals, one species).

### Host plants

A total of 14 families, 27 genera and 39 species amongst host plants of Cassidinae were collected in Longnan County Suppl. material 2.

Cassidinae larvae mainly feed on the plant families Poaceae, Rosaceae, Lamiaceae and Rubiaceae. Poaceae plants host 22 Cassidinae species, with nine species on *Miscanthus floridulus* (Labill.) Warb. ex K.Schum.

## Discussion

There are a growing number of reports on the species richness and diversity pattern of the Cassidinae beetle community, especially in Central and South America (Flowers and Hanson 2003, Wa̧sowska 2004, Freund 2004, Andrews and Gilbert 2005, Şen and Gök 2009, Şen and Gök 2014, Flinte et al. 2011, Flinte et al. 2009b, Staines 2011, Fernandes and Linzmeier 2012, Linzmeier and Ribeiro-Costa 2012, Sánchez-Reyes et al. 2019, Sánchez-Reyes et al. 2015, Ścibior et al. 2014, Ordóñez-Reséndiz et al. 2015, Magdalena et al. 2018, Niño-Maldonado et al. 2014, Niño-Maldonado et al. 2016, Flinte et al. 2009a, Simões and Monné 2011). However, no detailed community composition of Cassidinae beetles in China has been published. Even when we extended to other chrysomelid subfamilies, descriptions on leaf beetle diversity are still few (Li et al. 2011, Wu 2000a, Wu 2000b).

This study represented the first investigation on the community composition and species abundance of Cassidinae species in Longnan County and attempts to obtain a preliminary checklist of the Cassidinae species. To our knowledge, there are no similar analyses in China. The number of Cassidinae species collected in Longnan County accounted for approximately 11.7% of the national total (503). We found three newly recorded tribes in Jiangxi Province, with two tribes belonging to tortoise beetles (Suppl. material 1).

The earlier reports on Cassidinae host associations in China may have included many wrong records, due to misidentifications of either insects or plants or due to plants occasionally or incidentally being rested on by Cassidinae beetles. Here we tried to provide a relatively complete list of host plants, confirmed by the feeding damage of either larvae or adults. Most host plants were first reports for many Cassidinae beetle species. In our investigation, most Cassidinae species are oligophagous. For example, *Dactylispa xanthopus* Gestro feeds on *Rubus* spp. while *D. paucispina* Gressitt feeds on *Callicarpa* spp (Suppl. material 2).

All sampling sites showed that Hispini was richer than Cassidini in both species number and individual number. There are two possible reasons: (1) Generally, species number of Hispini (125) was higher than that of Cassidini (110) in China (Chen et al. 1986); (2) Cassidini mainly feed on the leaves of Dicotyledons, while Hispini feed on both Dicotyledons and Monocotyledons (Borowiec and Świętojańska 2019, Chen et al. 1986, Staines 2015). Some tribes were found in more abundance than others, which was not only happening in different sites, but also in the same site. One reason might be that different tribes had different number of species and the tribes with more species might have a higher chance of being discovered. The distribution of host-plants for different tribes is also uneven, some plants being much more common than others. The higher the number of individual plants, the more is the opportunity for the tribes to occur (i.e. plant apparency hypothesis) (Dai et al. 2017, Dai et al. 2018). Dominant and apparent plants are likely to host leaf-miners as a whole (Dai et al. 2018) or leaf-mining chrysomelids as a special case (Dai et al. 2017).

Amongst the three sites, seven tribes, 12 genera, 38 species and 422 individuals were collected at JLS; seven tribes, 14 genera, 37 species and 1047 individuals were collected at AJS; and only three tribes, six genera, 15 species and 121 individuals were collected at LGS. Moreover, all Cassidini were collected at AJS and JLS, while none was found at LGS. JLS had the highest number of Cassidinae species, which may due to its highest diversity in habitats and plants. For host plants of Cassidinae beetles, JLS was richest with 12 families, 22 genera and 28 species. AJS had 11 families, 20 genera and 26 species. LGS had only five families, nine genera and 12 species. It seemed that host plant richness decreased with the degree of human interference. The ecological environment at JLS is in a less disturbed condition, thus some sensitive Cassidinae species could survive (Fig. 2a, b). Some Cassidinae species preferred more disturbed forests (Fig. 2c, d), which might explain why AJS had the highest number of individuals. LGS was seriously modified by human activities and the number of plant species is fewest (Fig. 2e, f), which may explain its lower number of both species and individuals. Although we did not have the exact number of plant species in AJS and LGS, the faunistic diversity of Cassidinae might not be linearly correlated with plant diversity in the three sampling sites. However, low plant diversity in LGS definitely affected the occurrence and diversity of Cassidinae beetles. Compared to the cultivated LGS, the protected lands of JLS and AJS could not only provide more potential host plants, but also more diverse microhabitats, which might help to explain both fauna and abundance differences.

There are many ways to collect insects, including traps, nets, beating, smoking and manual searching. Each method has its advantages and disadvantages. A Malaise trap is suitable for collecting flying and crawling insects, but unsuitable for collecting jumping insects (Barney et al. 2007). Malaise traps have been used in some Cassidinae investigations (Chaboo 2012, Borowiec 2005, Barney et al. 2007, Chaboo and Staines 2015, Riley 2015, Fernandes and Linzmeier 2012). Malaise traps can gather many insect specimens by random and can collect for many years at a fixed site. It requires little time and labour (Flinte et al. 2009b). However, this method only yields a few Cassidinae adults, without biological and ecological information. Therefore, Malaise traps might not be appropriate to study insect-plant relationships and larval behaviour. A Light trap is suitable for long-term collecting of the insects with phototaxis (Wölfling et al. 2016, Mafia et al. 2018), but is not appropriate for collecting insects which are insensitive to light or cannot fly. Moreover, this method is only effective at night and would be affected by the full moon and weather and is sometimes limited by electricity availability (Hosking 1979). Light traps are not applicable for Cassidinae collecting because their phototaxis is weak and they are usually active in the daytime. Manual searching is convenient for nearly all insect groups. This method is also helpful to calrify the biology and ecology of insects, which might be hard to acquire by other methods. For Cassidinae, we can obtain much information about the adult, larva, pupa, host-plant, habitat and so on (Liao et al. 2018a, Liao et al. 2018b, Dai et al. 2014, Dai et al. 2013). Directed collecting (visual, sweeping and beating) obtained 50% of the hispine (s. str.) diversity at La Selva Biological Station, Costa Rica (Staines 2011). However, manual searching requires expertise knowledge on target insects and some species/individuals might be unintentionally ignored. The method is also time-consuming and labour-intensive. Therefore, manual searching might not be applicable for long-term monitoring and/or large-area investigation. Maybe the best way is to combine several methods together.

## Conclusions

Although the sample coverage curves indicate our data is complete enough to explain the species composition pattern in Longnan County, more detailed investigations, based on multiple collection methods, are still required for the analyses of temporal distribution and diversity-disturbance relationships. Moreover, we will try to perform such investigations in some typical regions in China, which may help to explain the geographical distribution, diversity pattern and host plant associations of Cassidinae.

## Supplementary Material

46910F0A-2EB6-54DC-817C-00A563B714B5 10.3897/BDJ.7.e39053.suppl1 Supplementary material 1 The Cassidinae beetles and their confirmed host plants at Longnan County, Jiangxi Province, China Data type: Table with identifications, host plants and occurrences
Brief description: Results of all identified Cassidinae beetles (mostly to species level, while a few to genus level) and their confirmed host plants (mostly to species level, while a few to genus level or family level), including the occurrences of Cassidinae species in three collection sites.
File: oo_340514.xlsx https://binary.pensoft.net/file/340514 Peng Liu, Chengqing Liao, Jiasheng Xu, Charles L Staines, Xiaohua Dai

904C81A7-D143-533E-9A1E-02E2D5595094 10.3897/BDJ.7.e39053.suppl2 Supplementary material 2 Host plants and their corresponding Cassidinae beetles at Longnan County, Jiangxi Province, China Data type: Table with occurrences
Brief description: Associations between host plants and their corresponding Cassidinae beetles.
File: oo_339931.xlsx https://binary.pensoft.net/file/339931 Peng Liu, Chengqing Liao, Jiasheng Xu, Charles L Staines, Xiaohua Dai

## Figures and Tables

**Figure 2a.**
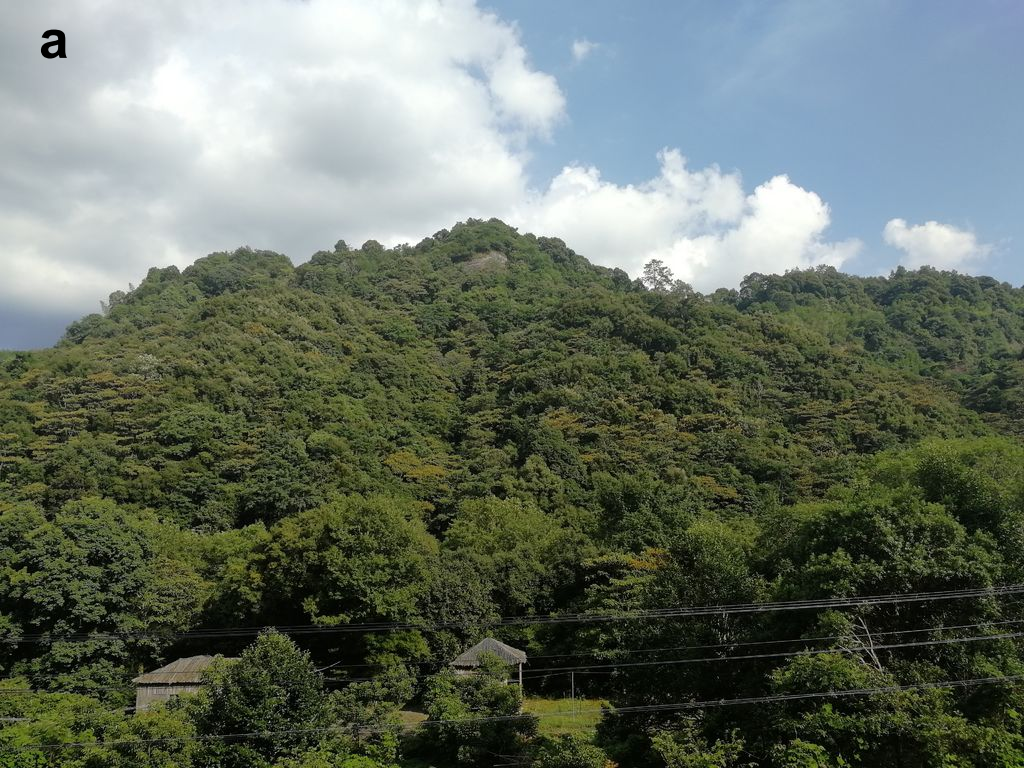
Jiulianshan

**Figure 2b.**
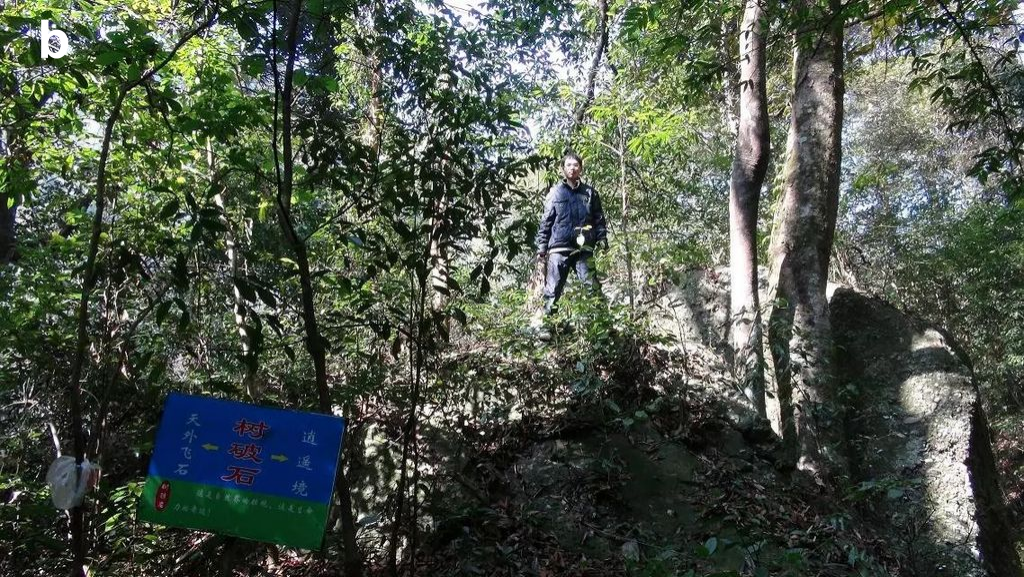
Jiulianshan

**Figure 2c.**
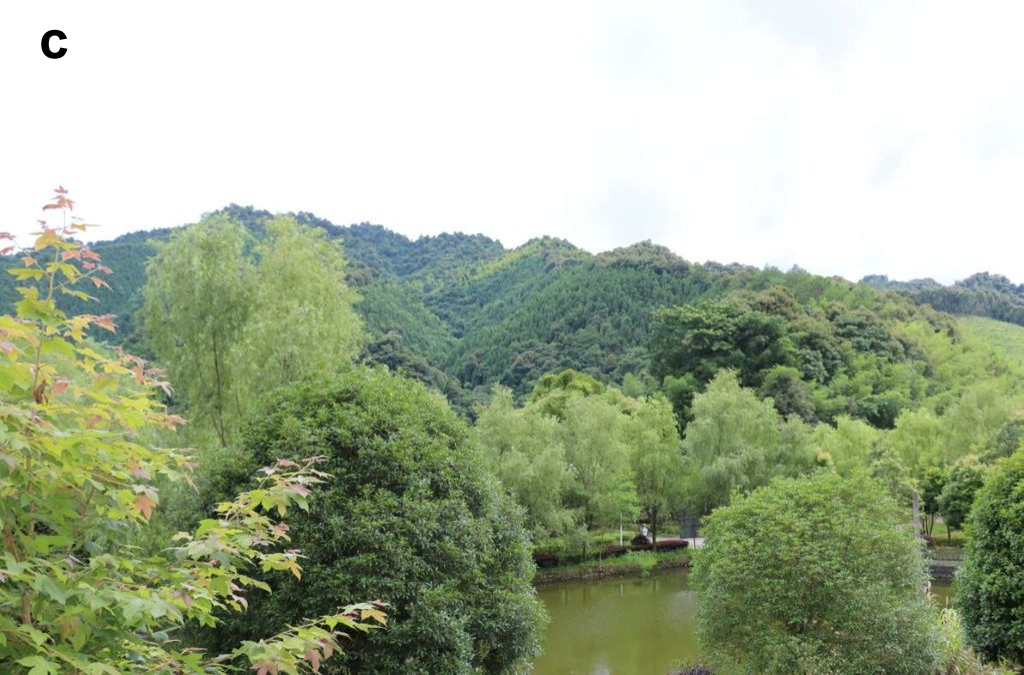
Anjishan

**Figure 2d.**
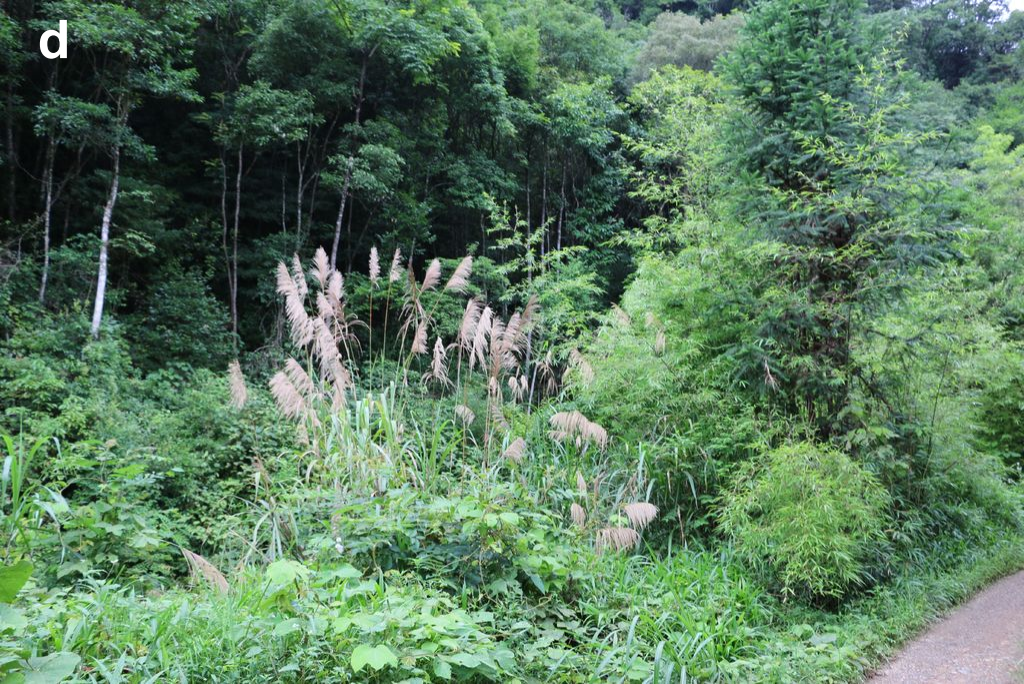
Anjishan

**Figure 2e.**
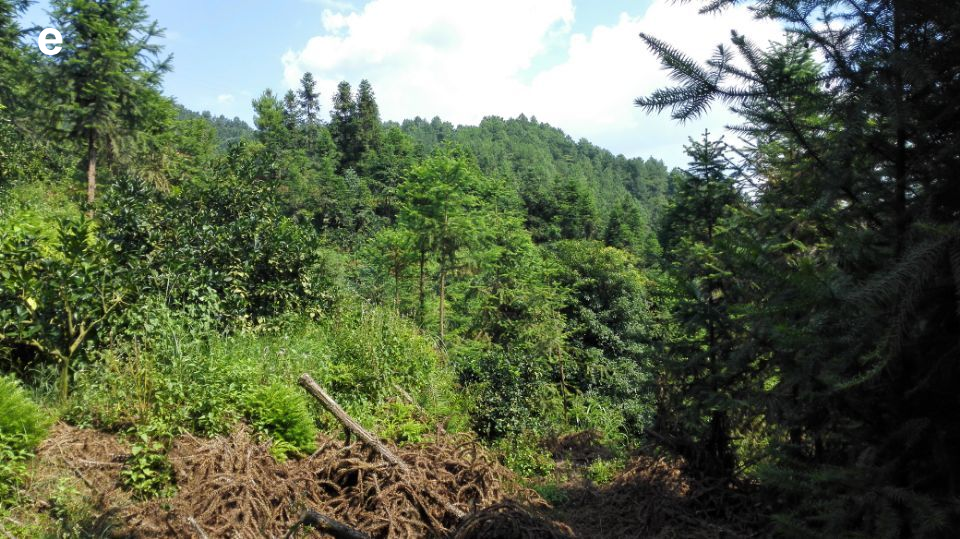
Leigongshan

**Figure 2f.**
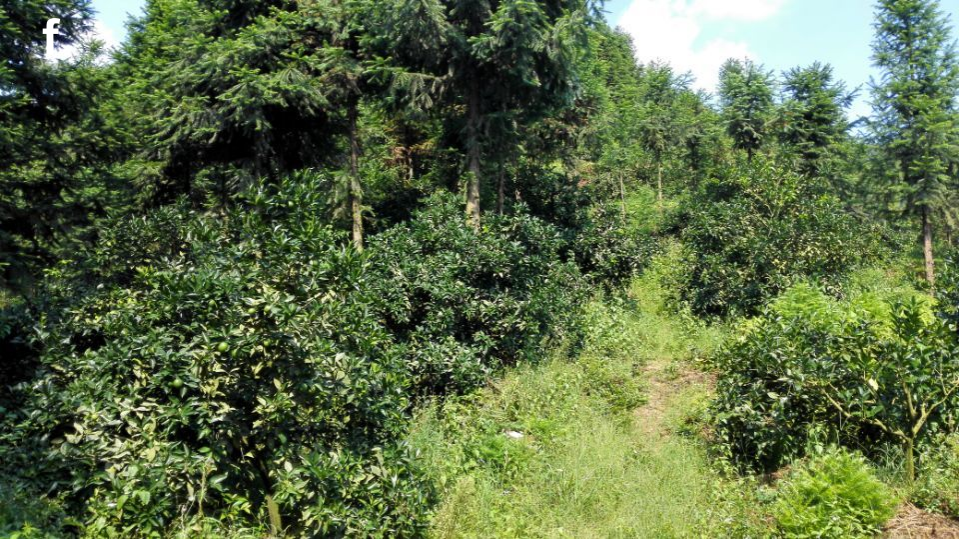
Leigongshan

**Figure 4a.**
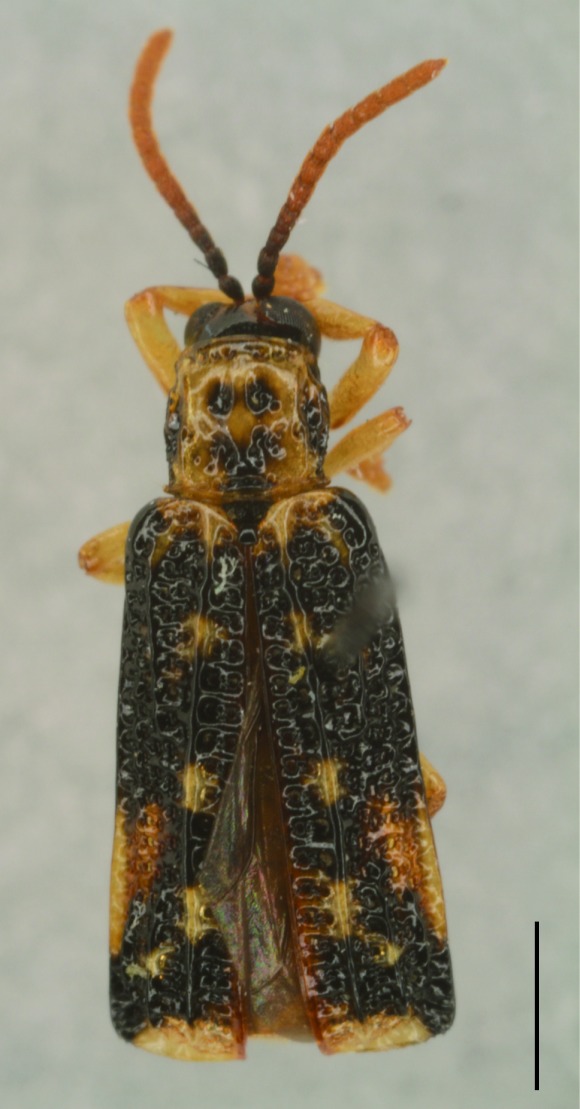
*Agonita foveicollis* Chen & Tan., Gonophorini.

**Figure 4b.**
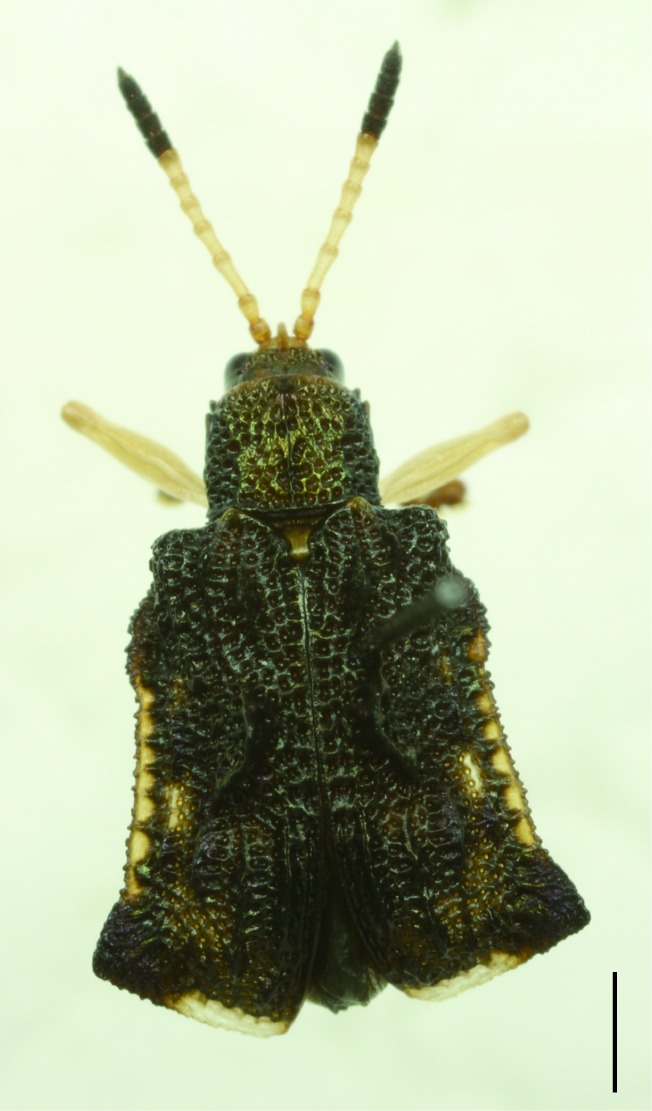
*Prionispa champaka* Maulik., Oncocephalini.

**Figure 4c.**
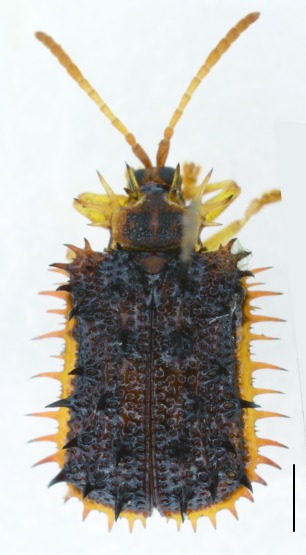
*Dactylispa maculithorax* Gestro., Hispini.

**Figure 4d.**
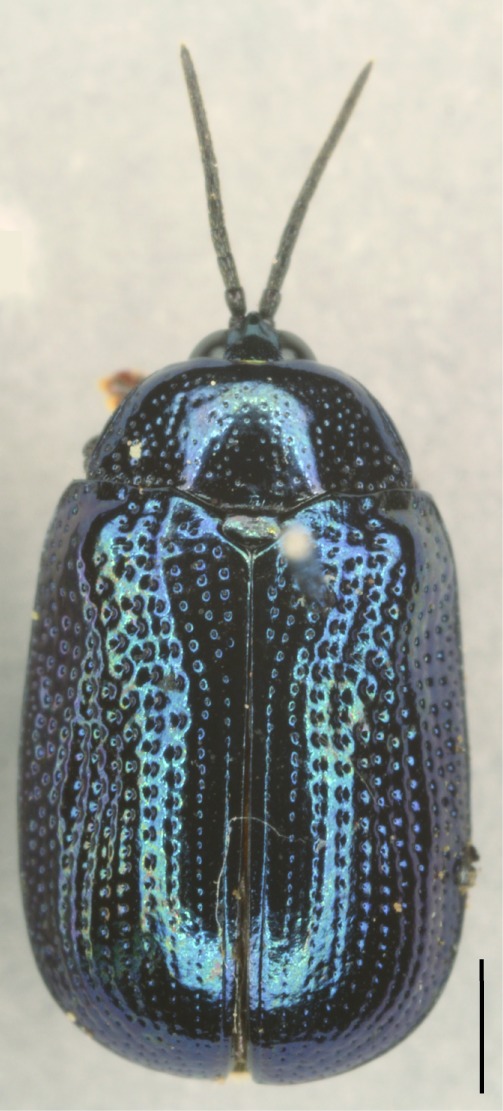
*Callispa bowringi* Baly., Callispini.

**Figure 5a.**
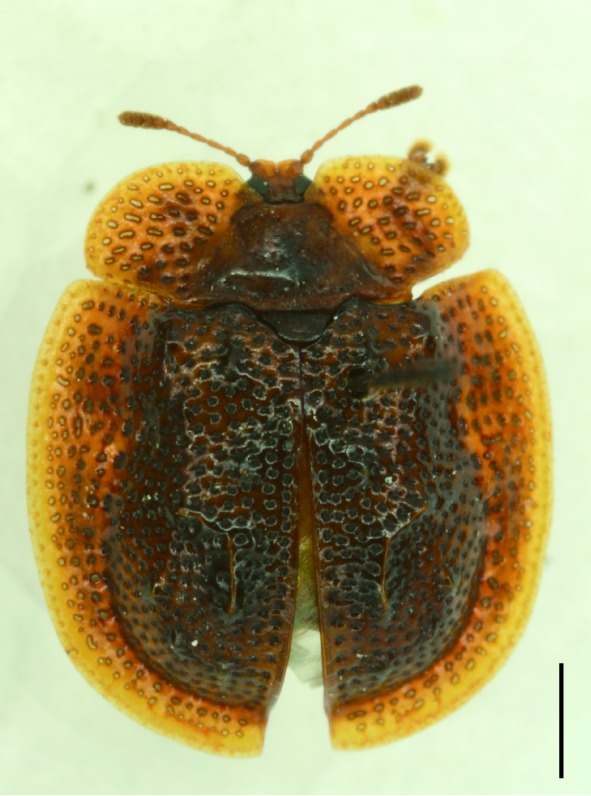
*Notosacantha sauteri* Spaeth., Notosacanthini.

**Figure 5b.**
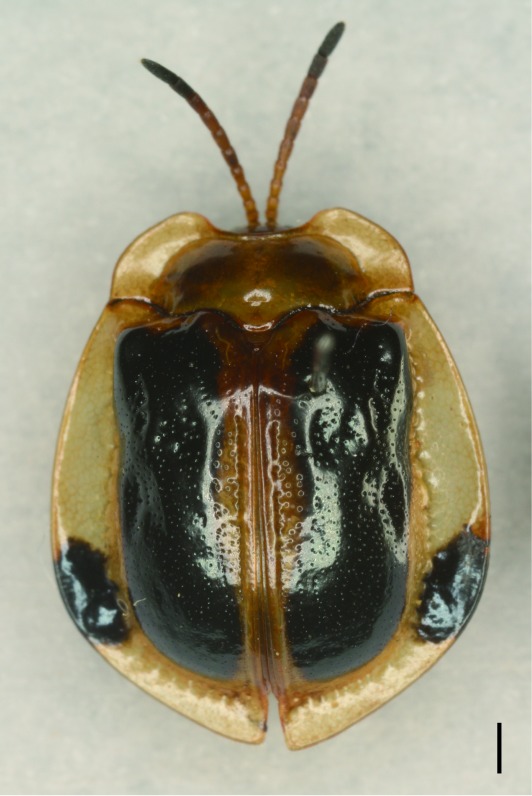
*Basiprionota whitei* Boheman., Basiprionotini.

**Figure 5c.**
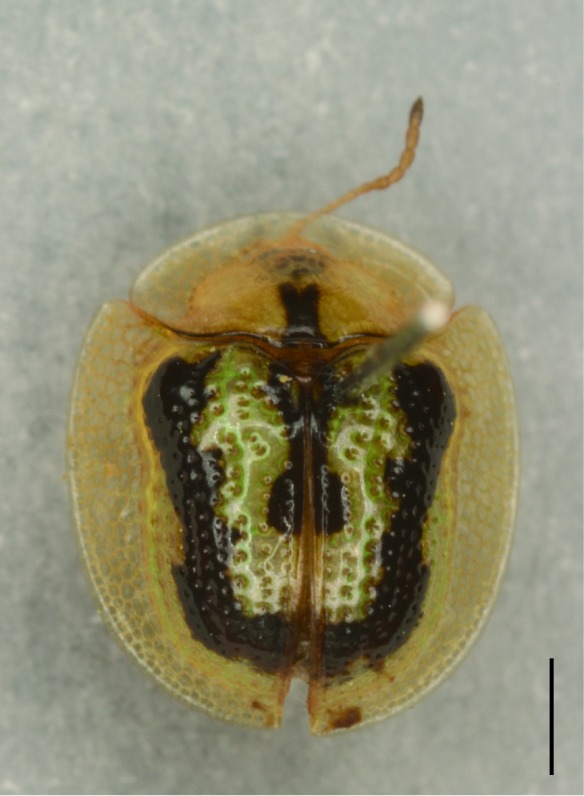
*Cassida circumdata* Herbst., Cassidini.

**Figure 5d.**
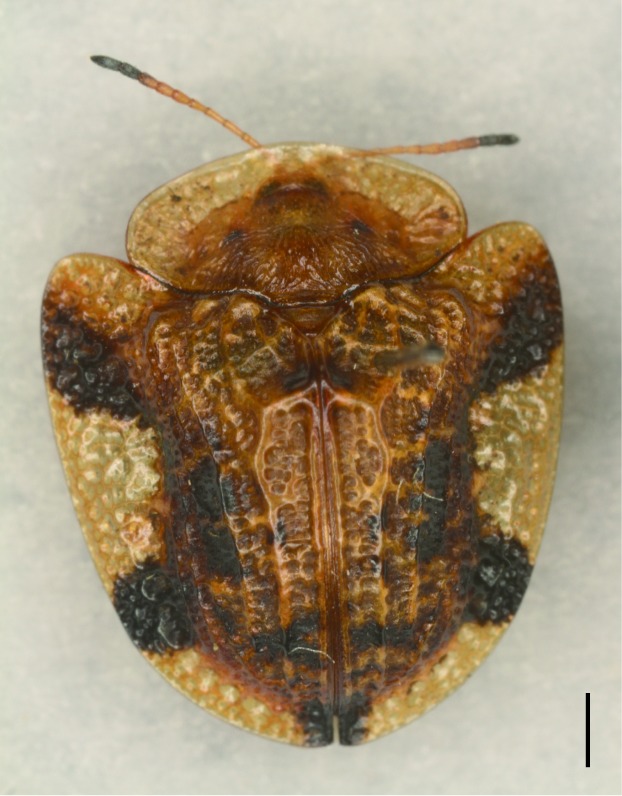
*Laccoptera nepalensis* Boheman., Aspidimorphini.
